# Transcriptomic Analysis of Blood Collagen-Induced Arthritis Mice Exposed to 0.1 THz Reveals Inhibition of Genes and Pathways Involved in Rheumatoid Arthritis

**DOI:** 10.3390/ijms252312812

**Published:** 2024-11-28

**Authors:** Mactar Ndiaga Dione, Qi Zhang, Sen Shang, Xiaoyun Lu

**Affiliations:** Key Laboratory of Biomedical Information Engineering of Ministry of Education, School of Life Science and Technology, Xi’an Jiaotong University, Xi’an 710049, China; mactardione@stu.xjtu.edu.cn (M.N.D.);

**Keywords:** terahertz wave, rheumatoid arthritis, hemocytes, transcriptomics, RNA sequencing

## Abstract

Inflammation plays an essential role in the phases of rheumatoid arthritis (RA) as the joints secrete a range of molecules that modulate the inflammatory process. While therapies based on physical properties have shown effectiveness in a range of animal experimental models, the understanding of their biological mechanisms remains unclear. The aim of this study was to investigate the immunomodulatory effects of a 0.1 terahertz (THz) wave in rheumatoid arthritis in an attempt to dissect the molecular pathways implicated. The collagen-induced rheumatoid arthritis (CIA) model joint mice were irradiated daily for 30 min over a period of 2 weeks with continuous 0.1 terahertz waves. High-throughput bulk RNA sequencing of the murine blood was performed to analyze and characterize the differences in gene expression changes between the control (Ctrl), CIA (RA), and CIA exposed to THz. Differentially expressed genes, canonical pathway analysis, gene set enrichment, and protein–protein interaction were further run on the selected DEGs. We found that terahertz exposure downregulated gene ontologies representing the “TGF-β signaling pathway”, “apoptosis”, “activation of T cell receptor signaling pathway”, and “non-canonical NF-κB signal transduction”. These observations were further confirmed by a decreased level in the expression of transcription factors *Nfib* and *Nfatc3*, and an increased level of *Lsp1*. In addition, the expression of *Mmp8* was significantly restored. These results indicate that THz ultimately attenuates the inflammatory response of hemocytes through the T cell and NF-κB pathway, and these changes are reverberated in the blood transcriptome. In this first report of transcriptome sequencing in a model of rheumatoid arthritis exposed to terahertz waves, the downregulated DEGs were associated with anti-inflammatory effects.

## 1. Introduction

The frequency spectrum of submillimeter terahertz (THz) electromagnetic irradiation occupies the frequency range immediately adjacent to the millimeter waves (MMWs) within the electromagnetic (EM) spectrum with a wavelength ranging from 0.01 to 3 mm. In the past few years, the mounting field of research as well as technological advancements in the THz frequency range have led to practical applications of electromagnetic radiation in the biomedical realm. The circumstantial evidence of perturbations in biosystems due to the vibrational and rotational energy levels of several biomolecules exhibits biological effects following THz exposure [1] and suggests that these complex perturbations are highly specific [2]. However, the lack of systematic studies hinders any further conclusive effects [3].

There are many bioeffects following THz exposure, irrespective of the material exposed [4]. Multiple studies describe cell proliferation, activation of stress reactions, and an increase in cell cytokines in a wide range of biological models [5]. Other responses include the establishment of reactive oxygen species (ROS), reactive nitrogen species (RNS), membrane destabilization, and enhancement of membrane permeability [6,7,8,9,10]. With regard to biosafety, it was hypothesized that, at a threshold under 0.15 THz at low-power density, THz exposure does not provoke any appreciable adverse reactions [1,11,12,13,14].

The current understanding of studies in human and murine models has revealed that THz exposure can inflate depression levels and inflammatory responses, and delay the wound healing response. It has also been shown to have effects on blood viscosity and erythrocyte deformability, aiding in the recovery of motor ability from spinal cord injury in mice, and alleviating cognitive dysfunction [5,15,16,17,18,19]. THz radiation is also thought to have curative properties and potential therapeutic usage. Electromagnetic radiation has the ability to demethylate numerous genes. The underlying mechanism that was hypothesized is that the excitation can disrupt methyl–DNA bridges. As THz irradiation is particularly absorbed by water, terahertz spectroscopy of blood cells is largely dependent on water, the main constituent of blood. In addition, distinct blood cells possess their own spectroscopic characteristics in the terahertz spectrum [13]. Decreased DNA methylation has been observed in vivo for blood cancer and melanoma cells lines following exposure to THz radiation [20,21]. It has even shown to have a benefit in downregulating genes involved in psoriasis [22]. Hyperthermia and general heat shock responses resulting from T-rays can also affect acute inflammatory reactions, which may trigger and precipitate restorative processes [23]. Combined with other treatments, THz shows promising results in speeding regeneration at wound and trauma sites and correction of pathophysiological disorders such as hypoxia and hypercoagulation [24,25]. In an observational investigation by Reukov et al. [26] on patients with ischemic stroke, acupuncture combined with infrared-THz radiation after six months was found to improve recovery. In a follow-up study, Kiryanova et al. [27] revealed that the application of THz-modulated infrared radiation enhanced cognitive function by restoring blood flow to the impacted hemisphere. This growing body of evidence provided valuable insights into the potential therapeutic applications of THz radiation.

The efficacy of physical therapies such as electric currents or electromagnetic fields at various frequencies, as a substitute for traditional pharmacological treatments, has been rigorously documented. They offer the advantages of being non-invasive and non-ionizing, stimulating collagen production [28], improving vascularization [29], and reducing pain [30]. The therapy may present shortcomings with regard to penetration depth, which might limit its effects for deep tissues and may necessitate several interventions to attain expected outcomes. The degree of THz penetration across the skin is not fully understood due to the unknown optical properties of the main constituent of the epidermis, melanin specifically. On top of that, various concentrations of melanin substantially affect the optical properties of the dermis. As opposed to the epidermis, the dermis holds high proportions of water, which diminishes the penetrability of THz waves [31,32].

Despite the challenges related to THz penetration and the need for further optimization of its delivery, its potential to modulate biological processes makes it an intriguing candidate for addressing inflammatory conditions. In particular, our previous studies have shown that 0.1 THz irradiation alleviated arthritis and exerted the immune-modulatory effects on CIA mice, while the mechanism of these effects on the immune system are still limited [33]. In the case of rheumatoid arthritis (RA), the inflammation is a physiological process entangled with both the innate and adaptative immune systems that occurs in response to the internal destruction of the joint. Numerous features may spark disruption in the joint, leading to disproportionate production of cytokines, ultimately causing an imbalance in the immune response homeostasis. Although RA strikes mainly the joints, the immune disorder may also impact organ systems such as the skin, bones, lungs, liver, heart, and eyes. Therefore, the use of alternative anti-inflammatory approaches such as TNF inhibitors, T cell co-stimulation inhibitors, and IL-6 inhibitors that interfere with the production of pro-inflammatory molecules is imperative for controlling inflammation [34,35]. As we revealed the therapeutic potential of THz radiation in CIA mice, it is essential to investigate its impact on the immune system at the cellular level through the profiles reflected by the changes in gene expression.

Building upon the relevance of the above-mentioned information, we performed RNA sequencing of white blood cells on a CIA mouse model. The CIA model is an arthritis-induced model in mice immunized with chicken type II collagen protein and sharing immunological and histological characteristics with RA. Currently, the CIA model is one of the gold standard mouse models for human RA, as it recreates the clinical symptoms of RA [36]. The CIA model provides an appropriate in vivo platform for the analysis of the effects of THz electromagnetic radiation on the immune system. We provide a comprehensive depiction of the workflow from the experimental design to data analysis of the transcriptome sequencing to investigate transcript-level differences and discern molecular pathway changes in response to 0.1 THz exposure in a rheumatoid arthritis model. One of the main novel findings is that terahertz exposure significantly shifts the expression of genes in the lipid metabolism and inflammatory pathways. These results will have certain value in guiding further rationale on the potential therapeutic effect of THz.

## 2. Results

### 2.1. Transcriptome Quality Assessment

The summary of the sequencing statistics and metrics including raw reads, clean reads, and mapped rate, is described in Table 1. Downstream analyses were undergone, using 242.489.052 (~242 million) clean reads. An average sequencing depth of ~40 million reads per sample was achieved, with an average percentage of Q30 of ~95.4%. All samples showed high throughput and sequencing quality and with the recommended number of reads, indicating reliability for further analyses.

### 2.2. Principal Component, Identification, and Analysis of DEGs

**Figure 1:**
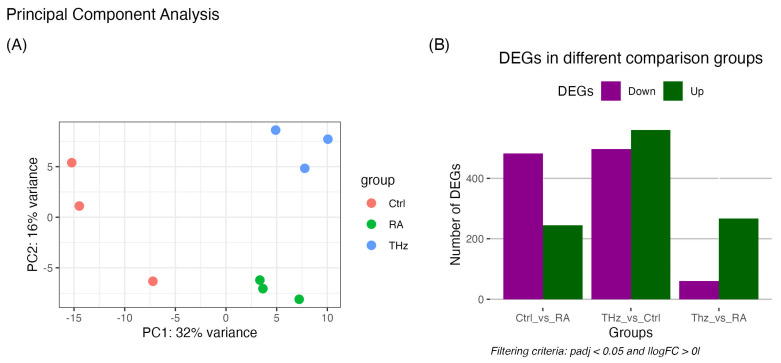

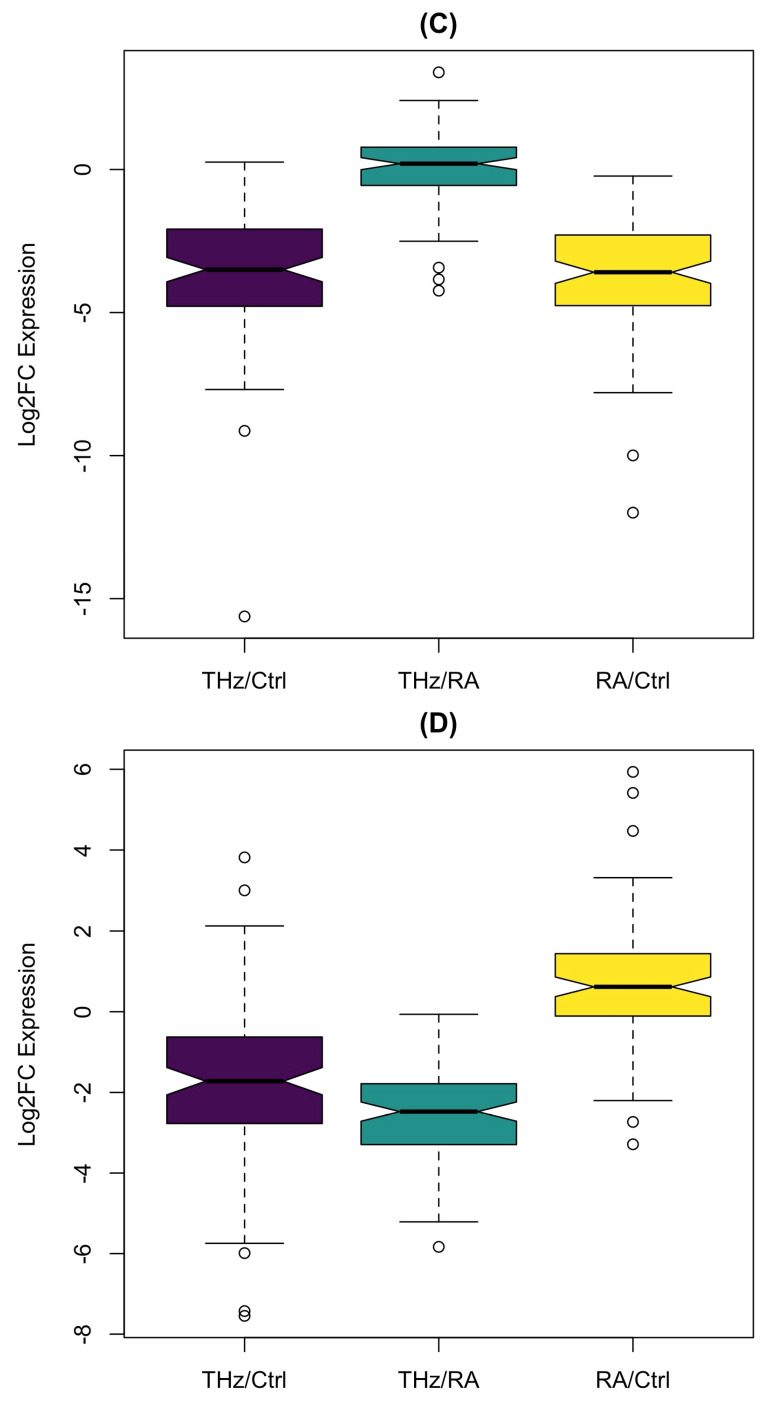
(**A**) PCA analysis of samples. Small circles represent samples as two-dimensional point, colored by conditions and biological replicate indicated by shape. The principal component (PCA1 and PCA2) accounted for 17–32% of variance in the dataset using the 500 most differentially expressed genes. The percentage values in the bracket of coordinate axis are ranked based on their magnitude to explain the variance. (**B**) Analysis of DEGs in different comparison groups: sham group, model group, and intervention group; (**C**,**D**) median of log2 fold change in PC1 and PC2, respectively.

To investigate the molecular mechanism of THz exposure in leukocytes, blood mRNA was extracted and transcriptomic analysis was applied to screen differentially expressed mRNA in three groups; control, CIA, and CIA + THz, (referred to as THz). The experimental design prioritized assessing the impact of THz radiation under disease conditions rather than in healthy mice. The principal component analysis (PCA) was performed on these three groups and the results are represented in Figure 1A. The plot allows us to briefly inspect the consistency and similarity patterns of expression within a group of biological replicates, as well as the dissimilarity between groups of replicates being compared. In this particular case, one cluster, exclusively the group of three biological replicates from control samples (shown in red), is properly segregated from the other cluster groups of six biological replicates from the disease model mice (shown in green and blue). More importantly, it can be observed at a glance, in the CIA groups, that the THz group is distinctly segregated from the other replicates of the CIA group.

The results of the DEGs screening between the three groups are shown in Figure 1B. The groups were screened for differential expression based on our set cut-off for padj and fold change criteria. In total, the pairwise comparisons resulted in 245 upregulated and 483 downregulated genes in the Control_vs_CIA group; 560 upregulated and 497 downregulated genes in the Control_vs_THz group and finally 267 upregulated and 60 downregulated genes in the THz_vs_CIA group. A list of the top 40 differentially expressed upregulated and downregulated genes between the CIA and THz groups is included as Appendix A data (Table A1 and Table A2) with their respective fold change values and adjusted *p*-values. The median of the log2 fold change was also calculated and is represented in Figure 1C,D. The notches in the plots show a very significant difference between the three groups pertaining to expression levels.

### 2.3. Clustering Analysis of Samples

**Figure 2:**
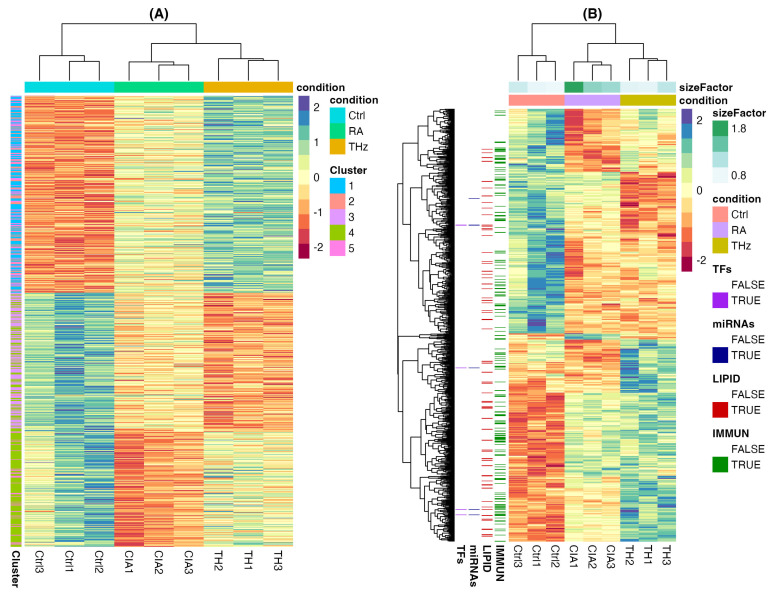
Transcriptional landscape of the different groups. (**A**) Optimized LRT heatmap representation of DEGs; (**B**) heatmap mapping GO terms to genes. Single row represents a gene and each column a sample. Colors indicated on the side represent the presence or absence of a gene in the three gene ontologies. Our data indicated that the shift in the transcriptome landscape was highly harmonized and regulated, clearly appearing specifically following THz irradiation.

For a comprehensive depiction of expression values, a map of the expression patterns of differentially expressed genes throughout individuals was inferred to shed light on clustering between samples and consistent expression patterns across genes. An unsupervised clustering analysis of averaged raw count values was performed. The resulting plot (Figure 2) shows the average relative expression of genes within three clusters and a conclusive shift in the hemocyte transcriptome in the form of upregulated and downregulated genes (*p* < 0.01; abs(LogFC) > 0). More importantly, a further mapping of these genes to their respective ontologies showed that they belonged to the immune system and the lipid metabolism and their profiles were disrupted (Figure 2B).

### 2.4. Enrichment Analysis of DEGs and Transcription Factor Prospection

**Figure 3:**
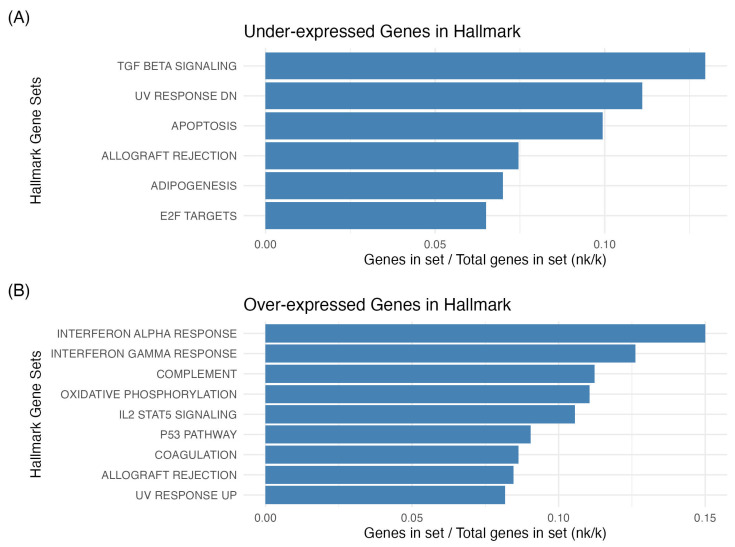
Diagram of enriched GO terms. The x-axis represents the ratio of genes in set over the total genes in set (nk/k), while the y-axis corresponds to the gene set in MsigDB. Under (**A**) and over-expressed genes (**B**).

**Figure 4:**
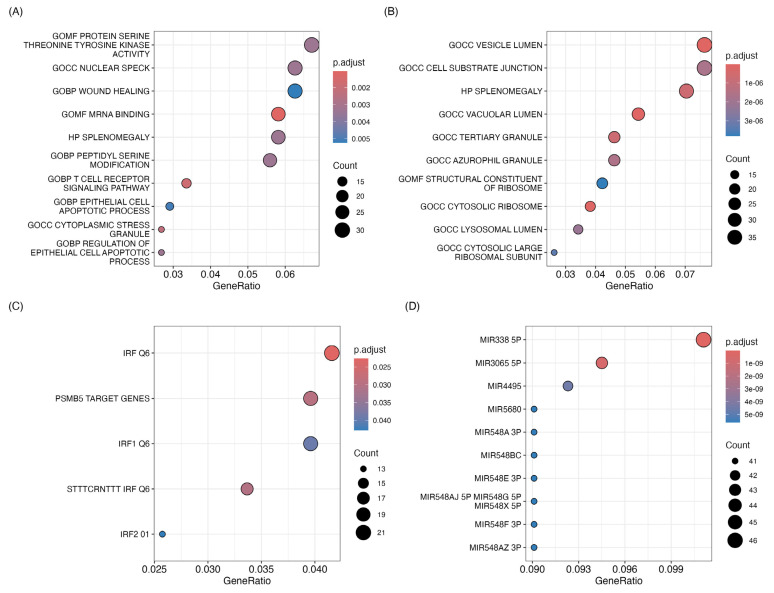
Functional analysis. Curated and canonical pathways: GO, KEGG-Reactome-Biocarta: under- (**A**) and over-expressed genes (**B**). Gene sets representing potential targets of regulation by transcription factors or microRNAs in under- and over-expressed genes (**C**,**D**).

Focusing on the effects of THz irradiation on CIA mice, we conducted further analysis on the differentially expressed genes in these two groups. The results of the functional analysis in Hallmark gene sets suggest that the differentially expressed genes were mainly enriched in TGF-β signaling, UV response, apoptosis, adipogenesis, and E2F targets for the downregulated genes (Figure 3A). For the upregulated genes, enrichment was found in interferon responses, complement, IL2 signaling, and other related pathways (Figure 3B). We also found that among the downregulated genes, there was enrichment in “wound healing”, and pathways related to signaling in leukocytes and apoptosis such as “T cell receptor signaling pathway”, “epithelial cell apoptotic process”, and “regulation of epithelial cell apoptotic process”. Despite the fact that upregulated genes accounted for a great proportion of the DEGs, their functional enrichment was all involved in cellular components and ribosome pathways. The 10 top pathways associated with the downregulated and upregulated genes are depicted in Figure 4A and 4B, respectively.

Furthermore, the transcription factor target screening from differentially expressed genes in the MsigDB C3 database resulted in distinct transcription factor targets in the downregulated genes, such as interferon regulatory factors (IRFs Q6), PSMB5 target genes, IRF1 Q6, STTTCRNTTT IRF Q6, and IRF2 (Figure 4C). Additionally, the analysis of the upregulated genes revealed significant enrichment of microRNAs such as miR-338-5p, miR-3065-5p, miR-4495, miR-5680, and miR-548A-3p (Figure 4D). 

To further explore the biological processes (BPs) involved, functional enrichment analysis with reference to Gene Ontology (GO) was performed using GOseq. We established a correlation between the genes differentially expressed and their associated functions. We identified significantly enriched GO modules (over-representation *p*-value < 0) in the list of differentially expressed genes and the screened candidate categories related to inflammation on the basis of a well-established role in the biological processes of interest are listed in Table 2.

### 2.5. Gene Set Enrichment Analysis of DEGs

We analyzed the important mutual pathways of interest through GSEA. Pathway analysis of the expression data revealed a range of potentially differentially enriched biological pathways in our exposed cells. Importantly, the result of GSEA showed that the following pathways (Regulation of Lipid Metabolic Process: Enrichment Score = 0.59; NES = 1.73; Lipid Catabolic Process: Enrichment Score = 0.64; NES = 1.89; Regulation of Protein Containing Complex: Enrichment Score = 0.56; NES = 1.72; Structural Molecule Activity: Enrichment Score = 0.52; NES = 1.63; Phagocytosis: Enrichment Score = 0.63; NES = 1.83; Negative regulation of Secretion: Enrichment Score = 0.70; NES = 1.89; Endocytic vesicle = Enrichment Score = 0.59; NES = 1.77) were particularly affected by THz, with a multitude of potential downstream implications (Figure S1).

The enrichment of key pathways like lipid metabolism, phagocytosis, and negative regulation of secretion from the DEGs suggested potential alterations in leukocytes metabolic reprogramming and attenuated immune response. It may reflect the relief of leukocytes activities after THz exposure, which could be critical areas for further exploration in understanding the underlying mechanisms of exposure effects.

### 2.6. Protein–Protein Interaction of DEGs

**Figure 5:**
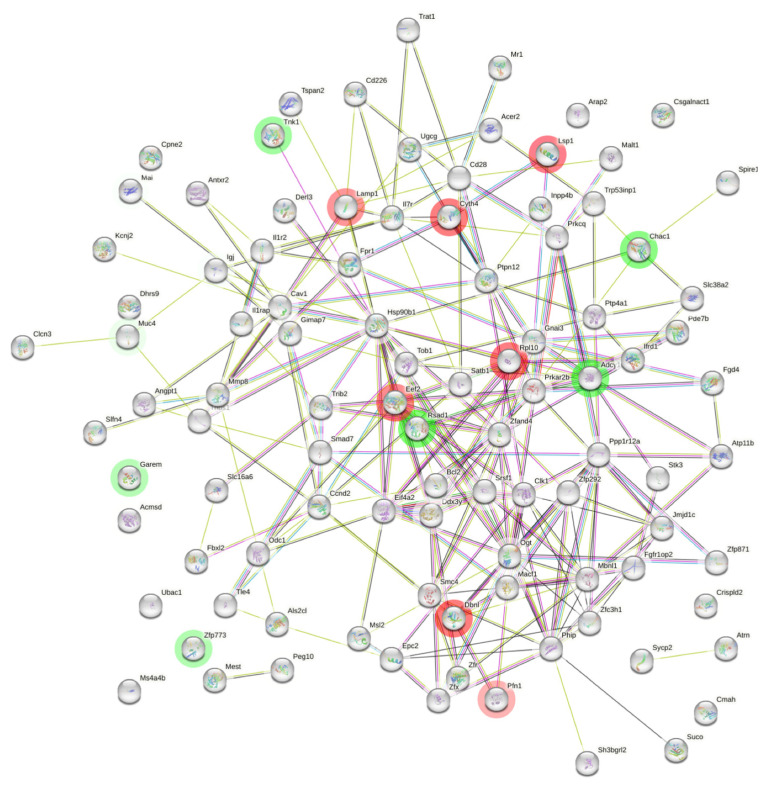
The nodes represent the genes (red = more expressed; green = less expressed) and the edges the type of interaction of the genes in the network. The edges have multiple colors depicting types of interaction.

The differentially expressed genes were used as input to build a PPI network. We constructed the network based on the magnitude of fold change of the selected genes which passed a threshold of *p* < 0.05. As shown in Figure 5, 223 interactions or edges were found against an expected number of 175 interactions, resulting in a significant enrichment of protein–protein interactions (*p*-value = 0.00026), thus displaying an important connectivity between these genes (Figure 5). We considered the highlighted genes in red (*Lsp1*, *Cyth4*, *Lamp1*, *Eef2*, *Rpl10*, *Dbnl*, *Pfn1*) and green (*Rsad1*, *Adyl1*, *Chac1*, *Tnk1*, *Garem1*, *Muc4*, and *Zfp773*) to have the highest and lowest LogFC in the PPI networks, respectively (*p* < 0.05). Direct or indirect evidence of the interactions among proteins is indicated by the density of the lines. The visualization of the PPI shed light on the complex molecular response triggered by THz exposure. 

### 2.7. Relative Expression of Genes Associated with Inflammatory Response and Lipid Metabolism

**Figure 6:**
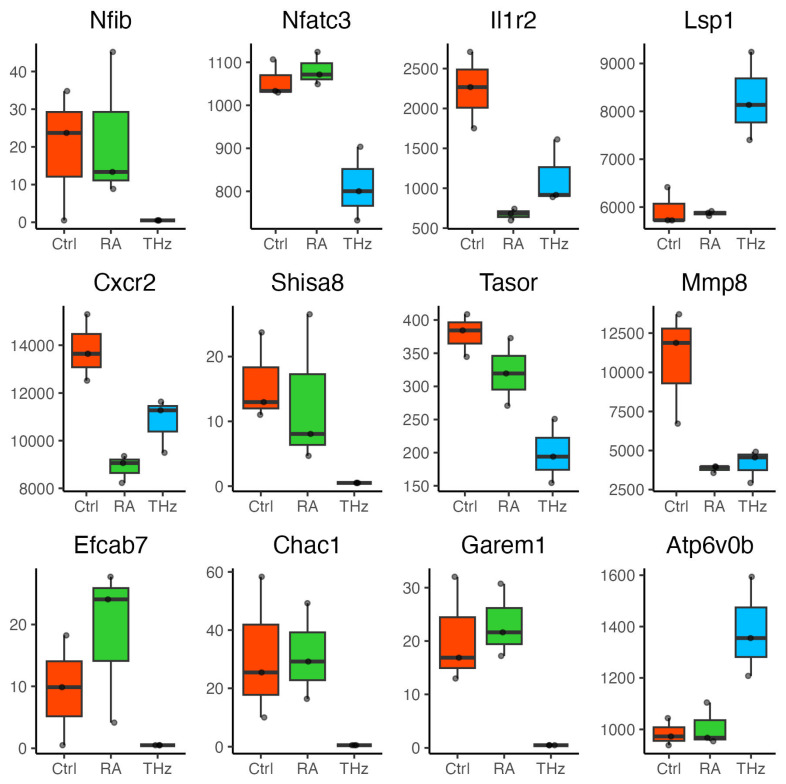
Boxplot of average expression of selected mRNA between the Ctrl in red, RA in green, THz groups in blue that showed a significant difference in expression.

According to the relative expressions, several genes associated with inflammatory response and lipid metabolism from the DEGs in the THz_vs_CIA group were carefully chosen based on their normalized count values and statistical significance. (*Nfib*, nuclear factor I/B, LogFC = −6.5135; *p* = 5.412909 × 10^−3^; *Nfatc3*, nuclear factor of activated T cells, cytoplasmic, calcineurin-dependent 3, LogFC = −6.5135; *p* = 5.412909 × 10^−3^; *Il1r2*, interleukin 1 receptor, type II, LogFC = −0.9772, padj = 6.080541 × 10^−4^; *Shisa8*, shisa family member 8 LogFC = −6.2067, *p* = 4.196414 × 10^−2^; *Mmp8*, matrix metallopeptidase 8, LogFC = −1.3797, *p* = 8.190396 × 10^−8^; *Efcab7*, EF-hand calcium binding domain 7 LogFC = −5.4224, *p* = 4.338837 × 10^−2^; *Chac1*, cation transport regulator 1, LogFC = −7.1916, *p* = 7.81 × 10^−5^; *Garem1*, GRB2-associated regulator of MAPK1 subtype 1, LogFC = −6.57, *p* = 3.728118 × 10^−4^; *Tasor*, transcription activation suppressor LogFC = −0.92, *p* = 3.047623 × 10^−3^; *Cxcr2*, C-X-C motif chemokine receptor 2 LogFC = −0.355, *p* = 0.03; *Atp6v0b*, ATPase, H+ transporting, lysosomal V0 subunit B LogFC = 0.4937, *p* = 1.383263 × 10^−2^; *Lsp1*, lymphocyte-specific 1 LogFC = 0.4721, *p* = 6.618633 × 10^−4^). Their expression levels were analyzed and are shown in Figure 6.

*Nfib* is a transcription factor regulated by the NF-κB pathway, and *Nfatc3* is typically associated with T cell activation. Decreased NF-kB consequently contributes to reducing inflammatory mediators in macrophages, neutrophils, and dendritic cells. In addition, the significant downregulation of these genes suggests that NF-κB signaling may be suppressed, leading to reduced T cell reactivity to antigens. *Cxcr2*, which is involved in neutrophil recruitment, shows decreased expression, indicating a potential reduction in neutrophil migration and associated inflammatory responses. *Mmp8*, a matrix metalloproteinase linked to inflammation and tissue remodeling, is also downregulated. Collectively, the downregulation of these genes implies that leukocytes may be undergoing a decrease in inflammatory signaling and immune activity.

## 3. Discussion

In recent years, NGS RNA sequencing and bioinformatics methods have become a comprehensive and sensitive technology to effectively answer a variety of biological queries with the ultimate goal of uncovering probable statistically differential transcripts [37,38]. The pathogenesis of RA is complicated and involves a persistent inflammatory response and over-expression of inflammatory mediators. Therefore, in the present study, we used next-generation sequencing analysis and reported the alterations in gene expression profiles in a mouse model of rheumatoid arthritis with the aim of divulging the main variations in the transcriptome in connection with repeated THz exposure. 

The blood transcriptome in control mice was different from that in the unirradiated CIA model and the CIA mice model exposed to THz. This result demonstrates the thoroughness and rigor of the mRNA sequencing data acquired from the samples. When compared to the other two groups, differential expression showed a low number of DEGs between CIA mice and CIA mice exposed to THz, mostly upregulated, suggesting a moderate rearrangement of the transcriptome in response to THz. Furthermore, clustering analysis of the commonly 712 differentially expressed mRNAs (*p* < 0.01) was transferred and displayed in a heatmap. A sharp alteration in the transcriptome landscape was revealed among these groups. The two heatmaps revealed that these DEGs have similar expression patterns within their respective groups, while having obviously different expression patterns between groups.

Pathway analysis of DEGs by the hypergeometric test revealed significant enrichment for numerous immune and inflammatory pathways. The expression of DEGs allowed us to identify a decrease in expression levels of genes mainly involved in the inflammatory response. Among the downregulated genes, the most prominent associations were the decreased expression of the participants in the “TGF-β signaling pathway”, “UV response”, and “apoptosis”. The multifunctional role of TGF-β and its subsequent effects on T cell population is still controversial in the pathogenesis of RA [39,40]. However, it has been argued that a blockade of TGF-β may decrease synovial cell proliferation, inflammation and angiogenesis, and modulate leukocytes and the magnitude of the immune response [41,42]. Apoptosis was also enriched in the downregulated genes. Apoptosis is considered to be a critical factor associated with RA [43]. The under-expression of the anti-apoptotic molecule *Bcl-2* (FC = −0.796367558, *p* = 6.19 × 10^−5^), which mediates cell death through caspase-9 and caspase-3, could be beneficial in RA [44]. 

The “T Cell Receptor Signaling pathway” showed distinct abundance in the downregulated genes, indicating a possible downregulation of the immune response to T cell subsets. T cell subsets play a distinct and critical role in the pathogenesis of RA [43]. CD4+ T helper cells, particularly Th17 cells, are key drivers of inflammation, promoting the production of pro-inflammatory cytokines such as IL-17, IL-6, and TNF-α. These cytokines contribute to synovial hyperplasia, bone erosion, and cartilage degradation. Regulatory T cells (Tregs), on the other hand, act to suppress excessive immune responses and maintain immune tolerance, though their function is often impaired in RA. Additionally, CD8+ T cells can accumulate in the joints, where they contribute to tissue damage by releasing cytotoxic molecules and pro-inflammatory cytokines. The enrichment of T cell-related signaling pathways from the blood leukocyte DEGs was well-coordinated with our previous experimental observation. The flow cytometry in Zhang et al. [33] showed a decrease in splenic T cells and a recovery to the original level of Treg cells, indicating a progressive improvement of the immune homeostasis. In addition, the level of IL-17, which promotes immune reaction and pro-inflammatory and bone-decaying cytokines, such as IL-1β, IL-6, and TNF-α, was reversed following 0.1 THz exposure in CIA mice. Moreover, although we did not have a group of THz-treated normal mice in this study, we did find that THz radiation reduced serum inflammatory factor levels in normal mice (data not published). Taken together, these findings suggest that THz radiation has an effectively beneficial role in inflammatory disease conditions, and T cells are an important group of cell populations that respond to THz irradiation.

In addition, we identified putative activation of “interferon alpha”, and “interferon gamma” responses, as well as “complement” and “IL2 stat signaling” in the upregulated genes. The expression levels of IFN-α, by promoting B cells, and the complement system change the plasma cell transcriptome towards a pro-inflammatory environment [45,46]. Interestingly, several interferon regulatory factors targets were also enriched from the downregulated genes. Although the detailed alterations and the underlying mechanisms are still waiting to be validated, these bioinformatic analysis results imply that the interferon signaling was significantly impacted by THz irradiation. It provided a potential objective for further investigation to reveal how THz radiation influences the function of blood leukocytes, thereby mitigating chronic inflammation in CIA mice. 

MicroRNAs are non-coding RNA molecules and play a substantial role in moderating non-coding RNAs. Among the upregulated genes, several miRNA targets, such as targets of miR-3065-5p and miR-548a-3p, were enriched. Previous studies have demonstrated that miR-548a-3p, which is significantly reduced in peripheral blood mononuclear cells (PBMCs) of RA patients, functions as a critical regulatory molecule in the pathogenesis of RA [47]. They implied that THz irradiation might also exert impacts on CIA mice by regulating various miRNAs, and their role in THz exposure studies needs to be given a particular interest as they may hide a relationship with regard to transcriptional activities.

To explore the biological processes (BPs) involved, GSEA was used to implement functional enrichment analysis with reference to Gene Ontology (GO). In particular, “Structural Molecule Activity”, “Regulation of Lipid metabolic process”, “regulation of protein containing complex assembly”, “Lipid catabolic process”, and “Negative regulation of secretion” pathways were identified as potentially altered by THz radiation. Identification of these pathways improves our comprehension of cellular mechanisms following THz exposure and sheds light on the genes responsible for the THz-induced responses in certain immunological disorders.

An extensive protein network based on co-expression was built for downregulated genes, in order to increase the chance of functional networks, thereby divulging the intricate multidimensional gene regulation mechanisms. There were strong connections between Mmp8, Il1rap, Il1r2, Il7r, HSp90b1, and Chac1. The network provided an overview of the major types of regulation observed, which may suggest that there is a tight biological connection among the genes. However, they were not exclusive.

A group of twelve DEGs was selected from our list of DEGs and reviewed in the literature regarding their role in auto-immune disorders, and their implications in RA. It is thought that over-expression of *Nfib* can promote the activation of NF-κB. Inhibition of the NF-κB pathway is one of the main potential therapeutic targets in the process of RA, as its level is significantly increased in the synovium and accelerates the development of RA. Activated NF-κB further induces translocation of NF-κB to the nucleus and facilitates the expression of targeted genes [43,48]. Due to its significantly reduced expression in the THz-exposed group compared to the other groups, one may speculate that this may cause the negative regulation of the NF-κB pathway observed in our enrichment results. Similar results have been reported by Toledano-Macìas et al. [35] in keratinocytes. The calcineurin-nuclear factor of activated T-lymphocytes (*Nfats*) is involved in many Ca^2+^/calmodulin-dependent pro-inflammatory pathways [49]. *Nfatc3*, in particular, is a transcriptional regulator of inflammatory genes in macrophages [50]. Control of Ca^2+^ influx is a key regulator for the activation and function of the adaptive immune response [51,52]. Diminished levels of Ca^2+^ in THz-exposed mice may suggest modulation of genes and pathways involved in the immune response. We noted a significant restoration in *Mmp8* expression levels in the THz-exposed group. *Lsp1* was also noted to be significantly increased in THz-exposed mice following exposure. A study by Hwang et al. [53] has shown that induction of T cells by Lps1 negatively regulated T cell migration by activation of the ERK pathway. The upregulation *Lsp1* genes in the blood translated into congruent modifications by regulation of T cells accumulation into inflamed joints. The tissue destruction characteristic of RA is the main source of production of metalloproteinases (Mmps) and joint destruction [54]. Collagenase-2 (*Mmp8*) deficiency in the RA model is reported to increase joint inflammation and bone erosion [55]. The immune function of *Cxcr2* in mice neutrophils is to regulate deployment of neutrophils and their migration from the bone marrow to the blood and ultimately, the inflammatory sites. Abrogation of *Cxcr2* amplifies delayed neutrophil survival in the joints and attenuates inflammatory arthritis [56,57]. THz exposure seems to increase its level.

## 4. Materials and Methods

### 4.1. Induction of RA Model and THz Source Intervention Methods

For the bioinformatics analysis, a total of nine healthy mice out of sixty in total with an average age of 7 weeks were acquired from GemPharmatech Co., Ltd. (Nanjing, China), housed as per standard procedures. CIA models were successfully established following the instructions in Miyoshi and Liu [58]. Mice were repeatedly injected with 0.1 mL equal volumes of CFA and Chick type II collagen and boosted three weeks after with collagen and IFA. Experiment procedures were cautiously refined to keep experimental animals’ suffering to a minimum.

THz source (0.1 THz, ~33 mW/cm^2^, TeraSense, San Jose, CA, USA) were used to radiate the mice ankle joints. To facilitate exposure, the animals were anesthetized with inhalation of isoflurane (RDW, Shenzhen, China). Mice were assigned into three groups of three individuals: non-irradiated mice used as control (sham) group (*n* = 3), one CIA animal model group (*n* = 3), and one CIA group treated with THz (*n* = 3). The animals of the latter group received 0.10 THz for a range exposure time of 30 min and taken back to their home cages during a period of two weeks.

### 4.2. RNA Extraction

Thereafter, peripheral blood was drained from the mice by venipuncture and RNA was isolated using TruSeq RNA Sample Preparation kit V2 (Illumina, San Diego, CA, USA). The integrity of the isolated RNA was evaluated to meet the required standards. RNA degradation and contamination were examined on 1% agarose gels. Nanodrop 2000 (Thermo Fisher Scientific, Waltham, MA, USA) was used to check the RNA purity. RNA purity is a crucial factor than can affect library preparation. Before the sequencing process, sample RNA integrity (RIN) and quality were assessed, by running a small amount on the Agilent 2100 bioanalyzer (Agilent Technologies, Santa Clara, CA, USA). Only high-quality RNA (OD 260/280 ≥ 2.0, RIN ≥ 6.5) was used for oligo-dT magnetic beads purification and mRNA sequencing.

### 4.3. RNA Quantification and Sequencing Library Construction

Following the quality control procedures, cDNA libraries were constructed from the nine samples with high-quality RNA according to the Illumina HiSeq 2500 platform (Illumina, San Diego, CA, USA). In a nutshell, an appropriate amount of total RNA was taken for mRNA enrichment using oligo-dT magnetic beads as a primer. After two rounds of magnetic bead purifications, thermal fragmentation was carried out at high temperature (94 °C) followed by cDNA synthesis. Library fragmentation into small pieces and purification were performed using the Agencourt AMPure XP-PCR Purification Beads (Beckman Coulter, Boston, MA, USA) to preferentially select sequences ranging from 100 pb to 300 pb in length. Thereafter, using fragments of mRNA, polymerase chain reaction (PCR) was performed with SuperScript IV Reverse Transcriptase (Thermo Fisher Scientific, Waltham, MA, USA). PCR products were further purified using Agencourt AMPure XP-PCR Purification Beads (Beckman Coulter, MA, USA). A Qubit 3.0 Spectrophotometer (Thermo Fisher Scientific, MA, USA) and Agilent 2100 bioanalyzer (Agilent Technologies, Santa Clara, CA, USA) were used to detect library concentration and library fragment length distribution, respectively. The library was finally pooled, then subjected to high-throughput sequencing on the Illumina platform in 2 × 150 pb paired-end sequencing.

### 4.4. Quality Control

In-house Perl scripts were first used to process the output reads. Raw sequence reads quality is a critical parameter in RNA sequencing pipelines. A general quality assessment of the raw sequences for each sample was first performed. The resulting raw FastQ reads for each sample were filtered and trimmed with Trim Galor (https://www.bioinformatics.babraham.ac.uk/projects/trim_galore/) (accessed on 5 November 2021) for removal of reads of low and bad quality and poly-N sequences. This allowed us to obtain a better alignment result in the later steps. Then, Q20 and Q30 were calculated.

### 4.5. Mapping Reads and Quantification of Gene Expression

The reads were then aligned to obtain transcript abundance. For alignment, we chose to map the reads against the whole mouse reference genome sequence to generate indexes. Using Hisat2 [59], a reference genome was built, and it processed paired-end reads mapped back to the same UCSC *Mus musculus* (mm10) mouse reference genome and reference assembled transcriptome. Quantification of mRNAs that overlap transcripts was performed by computing the raw counts value for every annotated gene for each sample. Then, the numbers of reads mapped reads to individual reference transcripts were counted by using StringTie (version 1.3.1) [60].

### 4.6. Differential Expression Analysis and Functional Profiling of DEGs

For significance screening, DESeq2 (version 1.42.1), a well-established and popular parametric tool, was chosen, as it has a comprehensive and explanatory manual user [61]. Hallmark (H) [62], Gene Ontology (GO) [63], Kyoto Encyclopedia for Genes and Genomes (KEGG) [64], Reactome [65], Pathways Interaction Database (PID) [66], and Biocarta [67] pathways were retrieved from the Molecular Signatures Database (MSigDB) (msigdb package version 7.5.1) for enrichment analysis of the differentially expressed genes using ClusterProfiler (version 4.10.1) [68] and GOseq (version 1.54.0) [69]. We restricted our core analyses to the considered interactions observed experimentally in *Mus musculus*. To run gene set enrichment analysis on these custom gene sets, we used the gene set enrichment analysis method (GSEA) from fgsea (version 1.28.0) [70] to detect pathways that were differentially modified by THz exposure and genes leading the enrichment. Similar to functional enrichment analysis, a second functional transcriptional regulation enrichment analysis for the systematic analysis of gene functions was performed in regulatory target gene sets for transcriptional regulators, specifically miRNAs and transcription factors. The interaction between genes was performed using the Search Tool for the Retrieval of Interacting Genes package (STRINdb, version 2.14.3) [71].

### 4.7. Statistical Analysis

As with all other statistical analyses performed, all methods used in this study were implemented in the R computing language (3.6.0/4.1.0) provided with the RStudio environment (http://www.r-project.org/, accessed on 10 December 2023). All packages are contained in the Bioconductor collection of R packages (http://www.bioconductor.org/about/, accessed on 10 December 2023) [72,73]. A false discovery rate *p* < 0.05 and a linear |log2 FoldChange| > 0 were adopted and set as thresholds for significantly differential expression genes unless otherwise stated.

## 5. Conclusions

In conclusion, this study utilized transcriptomic analysis to unveil the significant immunomodulatory effects of 0.1 THz radiation on a collagen-induced arthritis (CIA) mouse model, a representation of rheumatoid arthritis (RA). The identified DEGs and their functional analysis showed that immune and inflammatory-related genes and pathways, specifically the T cell and NF-κB signaling pathways, were involved in THz response following exposure. Moreover, GO and GSEA enrichment analyses also identified various inflammatory response-related biological processes as well as the alteration of the lipid metabolism pathway, suggesting the mitigation of inflammatory stress in blood leukocytes after 0.1 THz exposure. These findings, consistent with our previous works, corroborate the immunomodulatory virtue of THz irradiation, offering a molecular basis for the therapeutic effects of THz radiation. However, the extent and degree to which THz exposure affects immunosuppression and the potential regulatory mechanism proposed for these genes and pathways still require additional investigation. Therefore, this study paves the way for further research into the intersection of THz irradiation and immune regulation, ensuring broader relevance in the potential benefits of THz physical therapy for chronic inflammatory diseases.

## Figures and Tables

**Table 1:** Quality control statistics and sequencing information for samples.

Experimental Group	Raw Reads	Cleaned Reads	Q20 (%)	Q30 (%)	Total Mapped
Sham_1	25,319,706	24,223,564	98.5%	95.3%	95.7%
Sham_2	27,561,649	26,766,807	98.5%	95.2%	97.1%
Sham_3	25,528,610	24,802,746	98.6%	95.5%	97.2%
CIA_1	30,665,937	29,862,572	98.7%	95.7%	97.4%
CIA_2	25,947,320	25,098,704	98.5%	95.2%	96.7%
CIA_3	26,765,818	26,132,417	98.4%	95.0%	97.6%
CIA_1 + THz	26,352,525	25,687,839	98.6%	95.6%	97.5%
CIA_2+ THz	24,881,998	24,018,580	98.6%	95.5%	96.5%
CIA_3+ THz	29,465,489	28,181,960	98.6%	95.6%	95.6%

[1] Sham_1, 2, and 3 are cDNA libraries of Sham group, CIA group (CIA_1, 2, 3), CIA + THz group (CIA_1 + THz, CIA_2 + THz, CIA_3 + THz); [2] Raw reads: count number of original sequence data; [3] Clean reads: count number of sequencing data after filtering; [4] Q20% and Q30%: count Phred values; [5] Total mapped: number of clean reads that can be found on the genome.

**Table 2:** Inflammation-related ontologies screened based on enrichment in GOseq THz_vs_CIA.

Category	Over_ Represented_ *p* Value	Term	Ontology
GO:0033007	0.00331871	negative regulation of mast cell activation involved in immune response	BP
GO:0002887	0.00354279	negative regulation of myeloid leukocyte-mediated immunity	BP
GO:0042060	0.00580161	wound healing	BP
GO:1901223	0.00612836	negative regulation of non-canonical NF-kappaB signal transduction	BP
GO:0006954	0.00735966	inflammatory response	BP
GO:0002283	0.01093672	neutrophil activation involved in immune response	BP
GO:0032692	0.0135862	negative regulation of interleukin-1 production	BP
GO:1905460	0.01556618	negative regulation of vascular-associated smooth muscle cell apoptotic process	BP
GO:0001817	0.01562121	regulation of cytokine production	BP
GO:1905258	0.0178608	regulation of nitrosative stress-induced intrinsic apoptotic signaling pathway	BP
GO:1905259	0.0178608	negative regulation of nitrosative stress-induced intrinsic apoptotic signaling pathway	BP
GO:0002704	0.02029447	negative regulation of leukocyte-mediated immunity	BP
GO:0033177	0.01314609	proton-transporting two-sector ATPase complex, proton-transporting domain	CC
GO:0005637	0.03431904	nuclear inner membrane	CC
GO:0034364	0.03550117	high-density lipoprotein particle	CC
GO:0034358	0.0440191	plasma lipoprotein particle	CC
GO:1990777	0.0440191	lipoprotein particle	CC
GO:0009986	0.04494883	cell surface	CC
GO:0061134	0.00049736	peptidase regulator activity	MF
GO:0016620	0.00263277	oxidoreductase activity, acting on the aldehyde or oxo group of donors, NAD or NADP as acceptor	MF
GO:0008289	0.0031631	lipid binding	MF
GO:0019966	0.01722421	interleukin-1 binding	MF
GO:0005011	0.02783496	macrophage colony-stimulating factor receptor activity	MF
GO:0034987	0.02961874	immunoglobulin receptor binding	MF
GO:0070325	0.03676257	lipoprotein particle receptor binding	MF

## Data Availability

The raw transcriptome sequencing data discussed in this study have been submitted to the Gene Expression Omnibus (GEO) GSE77499.

## Supplementary Materials

The following supporting information can be downloaded at: https://www.mdpi.com/article/10.3390/ijms252312812/s1.

## Appendix A

**Table A1:** Top 40 under-expressed genes between THz_vs_CIA.

Symbol	Gene Name	log2FoldChange	padj
*Jchain*	immunoglobulin joining chain	−3.549513824	6.59 × 10^−51^
*Crispld2*	cysteine-rich secretory protein LCCL domain-containing 2	−2.115560042	8.33 × 10^−17^
*Il7r*	interleukin 7 receptor	−0.871448645	2.61 × 10^−11^
*Cd28*	CD28 antigen	−1.188825796	6.15 × 10^−11^
*Trp53inp1*	Transformation-related protein 53-inducible nuclear protein 1	−0.86053661	1.64 × 10^−8^
*Macf1*	microtubule-actin crosslinking factor 1	−0.770775484	2.09 × 10^−8^
*Peg10*	paternally expressed 10	−1.697575125	2.69 × 10^−8^
*Kcnj2*	potassium inwardly rectifying channel, subfamily J, member 2	−1.020747308	5.01 × 10^−8^
*Acer2*	alkaline ceramidase 2	−0.967994966	7.65 × 10^−8^
*Mmp8*	matrix metallopeptidase 8	−1.37971112	8.19 × 10^−8^
*Cmah*	cytidine monophospho-N-acetylneuraminic acid hydroxylase	−0.702814914	1.66 × 10^−7^
*Mr1*	major histocompatibility complex, class I-related	−1.276531767	2.02 × 10^−7^
*Cd226*	CD226 antigen	−1.139836494	4.25 × 10^−7^
*Smc4*	structural maintenance of chromosomes 4	−0.872094502	4.93 × 10^−7^
*Mest*	mesoderm-specific transcript	−1.08564923	5.66 × 10^−7^
*Acmsd*	amino carboxymuconate semialdehyde decarboxylase	−1.826592753	1.75 × 10^−6^
*Inpp4b*	inositol polyphosphate-4-phosphatase, type II	−1.044687259	1.83 × 10^−6^
*Hsp90b1*	heat shock protein 90, beta (Grp94), member 1	−0.838853217	2.79 × 10^−6^
*Zfand4*	zinc finger, AN1-type domain 4	−1.007212229	3.51 × 10^−6^
*Eif4a2*	eukaryotic translation initiation factor 4A2	−0.716832344	4.04 × 10^−6^
*Slc38a2*	solute carrier family 38, member 2	−0.776320758	4.89 × 10^−6^
*Dhrs9*	dehydrogenase/reductase 9	−0.979717992	7.62 × 10^−6^
*Trat1*	T cell receptor-associated transmembrane adaptor 1	−1.277897079	8.24 × 10^−6^
*Zfx*	zinc finger protein X-linked	−1.090494625	8.57 × 10^−6^
*Ugcg*	UDP-glucose ceramide glucosyltransferase	−0.874102244	9.61 × 10^−6^
*Fpr1*	formyl peptide receptor 1	−1.017961232	1.05 × 10^−5^
*Antxr2*	anthrax toxin receptor 2	−0.69226448	1.51 × 10^−5^
*Ogt*	O-linked N-acetylglucosamine (GlcNAc) transferase (UDP-N-acetylglucosamine:polypeptide-N-acetylglucosaminyl transferase)	−0.751332018	1.51 × 10^−5^
*Muc4*	mucin 4	−5.20559844	1.72 × 10^−5^
*Stk3*	serine/threonine kinase 3	−1.186962537	2.07 × 10^−5^
*Angpt1*	angiopoietin 1	−0.842783152	2.55 × 10^−5^
*Csgalnact1*	chondroitin sulfate N-acetylgalactosaminyltransferase 1	−1.505463396	3.22 × 10^−5^
*Eef2*	eukaryotic translation elongation factor 2	0.462766835	3.27 × 10^−5^
*Spire1*	spire type actin nucleation factor 1	−1.223773761	3.35 × 10^−5^
*Il1rap*	interleukin 1 receptor accessory protein	−0.893550461	3.44 × 10^−5^
*Mbnl1*	Muscleblind-like splicing regulator 1	−0.662795058	3.65 × 10^−5^
*Satb1*	special AT-rich sequence binding protein 1	−0.646083964	4.91 × 10^−5^
*Als2cl*	ALS2 C-terminal like	−1.258857474	5.39 × 10^−5^
*Bcl2*	B cell leukemia/lymphoma 2	−0.796367558	6.19 × 10^−5^
*Suco*	SUN domain-containing ossification factor	−1.07688349	7.72 × 10^−5^

**Table A2:** Top 40 over-expressed genes between THz_vs_CIA.

Symbol	Gene Name	log2FoldChange	padj
*Apoe*	apolipoprotein E	1.869284605	2.30 × 10^−48^
*Camp*	cathelicidin antimicrobial peptide	4.649222816	5.10 × 10^−25^
*Chil3*	chitinase-like 3	1.464223072	6.96 × 10^−24^
*Tifab*	TRAF-interacting protein with forkhead-associated domain, family member B	1.782294357	1.01 × 10^−20^
*Tmem176b*	transmembrane protein 176B	2.807758186	1.56 × 10^−20^
*Fn1*	fibronectin 1	1.645821302	3.51 × 10^−20^
*Psap*	prosaposin	0.813929991	1.09 × 10^−17^
*S100a4*	S100 calcium binding protein A4	1.341661792	5.16 × 10^−17^
*Csf1r*	Colony-stimulating factor 1 receptor	1.065731925	1.71 × 10^−16^
*Lyz2*	lysozyme 2	0.796185491	7.21 × 10^−16^
*Ctss*	cathepsin S	1.132059628	5.17 × 10^−15^
*Cybb*	cytochrome b-245, beta polypeptide	1.168899553	5.17 × 10^−15^
*Ltf*	lactotransferrin	4.325758455	8.42 × 10^−15^
*Tmem176a*	transmembrane protein 176A	2.660399007	5.25 × 10^−14^
*Ms4a6d*	membrane-spanning 4-domains, subfamily A, member 6D	1.749086124	2.23 × 10^−13^
*Lrp1*	low density lipoprotein receptor-related protein 1	1.721304531	2.44 × 10^−13^
*Plac8*	placenta-specific 8	1.611941032	1.58 × 10^−12^
*Ifitm3*	Interferon-induced transmembrane protein 3	1.166726477	2.20 × 10^−12^
*Grn*	granulin	0.993091255	1.29 × 10^−10^
*Plxnd1*	plexin D1	1.406893256	2.10 × 10^−9^
*Ptpro*	protein tyrosine phosphatase receptor type O	1.482162376	2.33 × 10^−9^
*Pld4*	phospholipase D family member 4	1.309314223	6.37 × 10^−9^
*Ifi30*	interferon gamma-inducible protein 30	1.090629815	9.32 × 10^−9^
*Rack1*	receptor for activated C kinase 1	0.647134613	9.81 × 10^−9^
*Vim*	vimentin	0.657953058	1.01 × 10^−8^
*Ctsa*	cathepsin A	1.05154966	1.27 × 10^−8^
*Serpina3g*	serine (or cysteine) peptidase inhibitor, clade A, member 3G	2.174397334	1.35 × 10^−8^
*Ctsh*	cathepsin H	0.942888264	2.07 × 10^−8^
*Fcgr1*	Fc receptor, IgG, high affinity I	2.583841986	2.25 × 10^−8^
*Myof*	myoferlin	2.779352002	2.48 × 10^−8^
*Ngp*	neutrophilic granule protein	3.139842336	4.30 × 10^−8^
*Fcgrt*	Fc fragment of IgG receptor and transporter	1.359766608	5.09 × 10^−8^
*Lst1*	leukocyte-specific transcript 1	1.011173533	5.87 × 10^−8^
*Krt80*	keratin 80	1.12369742	9.74 × 10^−8^
*Cfh*	complement component factor h	1.695515037	1.13 × 10^−7^
*Rassf4*	Ras association (RalGDS/AF-6) domain family member 4	1.139565148	1.19 × 10^−7^
*B4galnt1*	beta-1,4-N-acetyl-galactosaminyl transferase 1	0.748656064	1.19 × 10^−7^
*Clec12a*	C-type lectin domain family 12, member a	1.1889603	1.97 × 10^−7^
*Gatm*	glycine amidinotransferase (L-arginine:glycine amidinotransferase)	2.079182928	4.25 × 10^−7^
*Arhgef37*	Rho guanine nucleotide exchange factor 37	2.340004741	4.93 × 10^−7^
