# Supplementary material for: Transcriptomic Analysis of Blood Collagen-Induced Arthritis Mice Exposed to 0.1 THz Reveals Inhibition of Genes and Pathways Involved in Rheumatoid Arthritis
Source: Int J Mol Sci. 2024 Nov 28;25(23):12812. doi: 10.3390/ijms252312812 (PMC11640783; doi:10.3390/ijms252312812)
Supplement: Supplementary file 1 [file ijms-25-12812-s001.zip › ijms-3283865-supplementary.pdf]

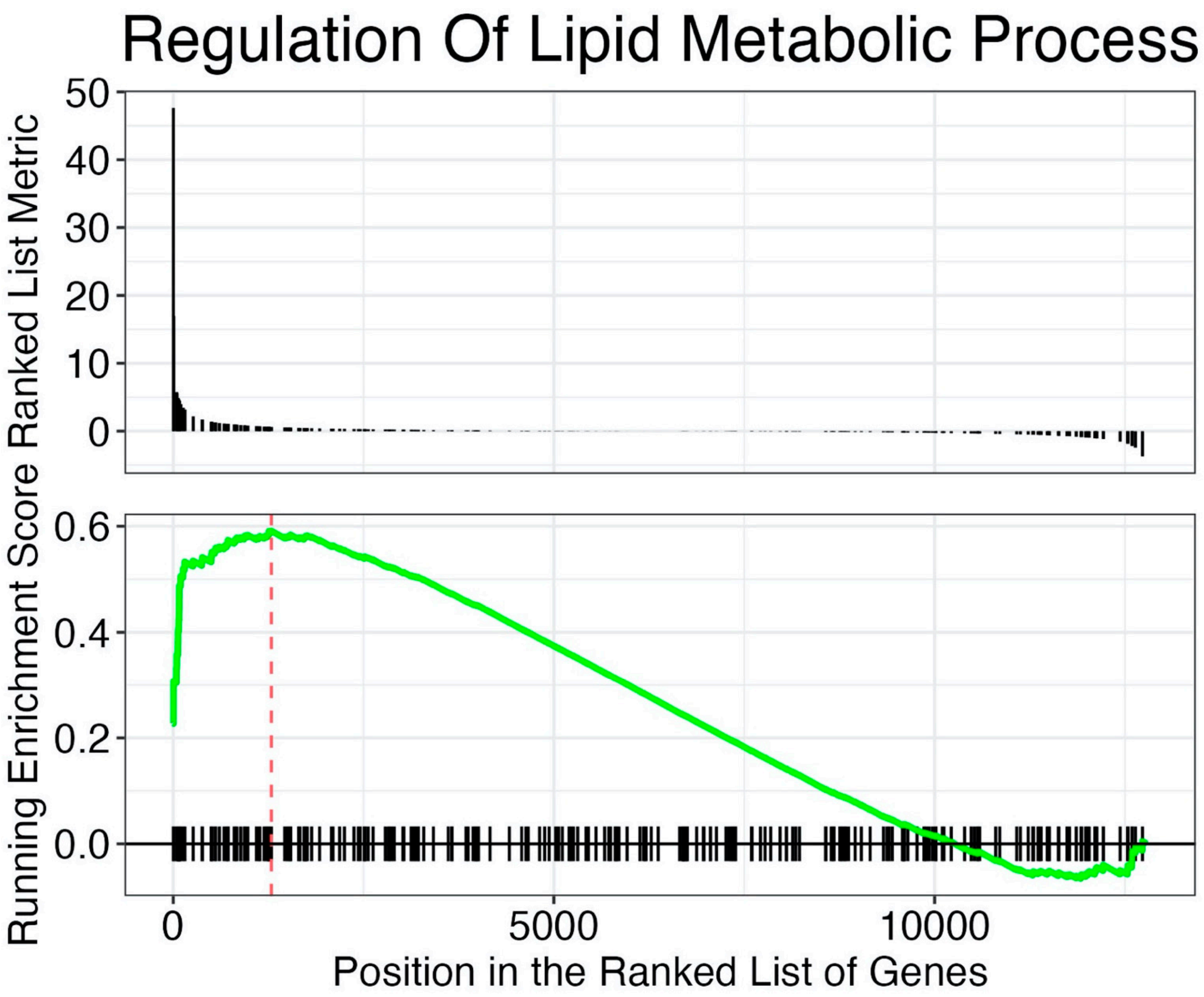
Regulation Of Lipid Metabolic Process
Ranked List Metric
50
40
30
20
10
0
Running Enrichment Score
0.6
0.4
0.2
0.0
0
5000
10000
Position in the Ranked List of Genes

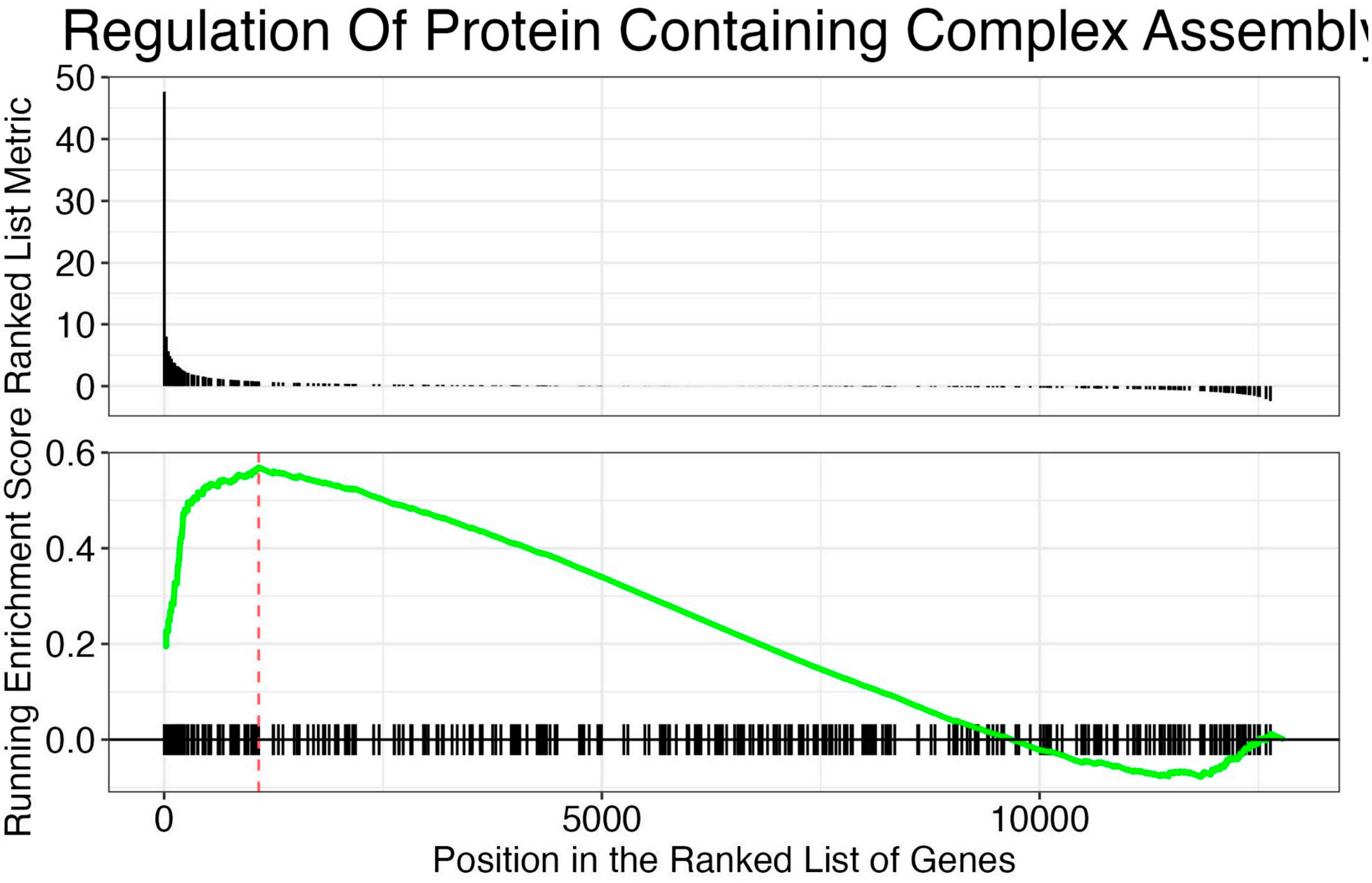
Regulation Of Protein Containing Complex Assembly
Running Enrichment Score Ranked List Metric
50
40
30
20
10
0
0.6
0.4
0.2
0.0
0
5000
10000
Position in the Ranked List of Genes

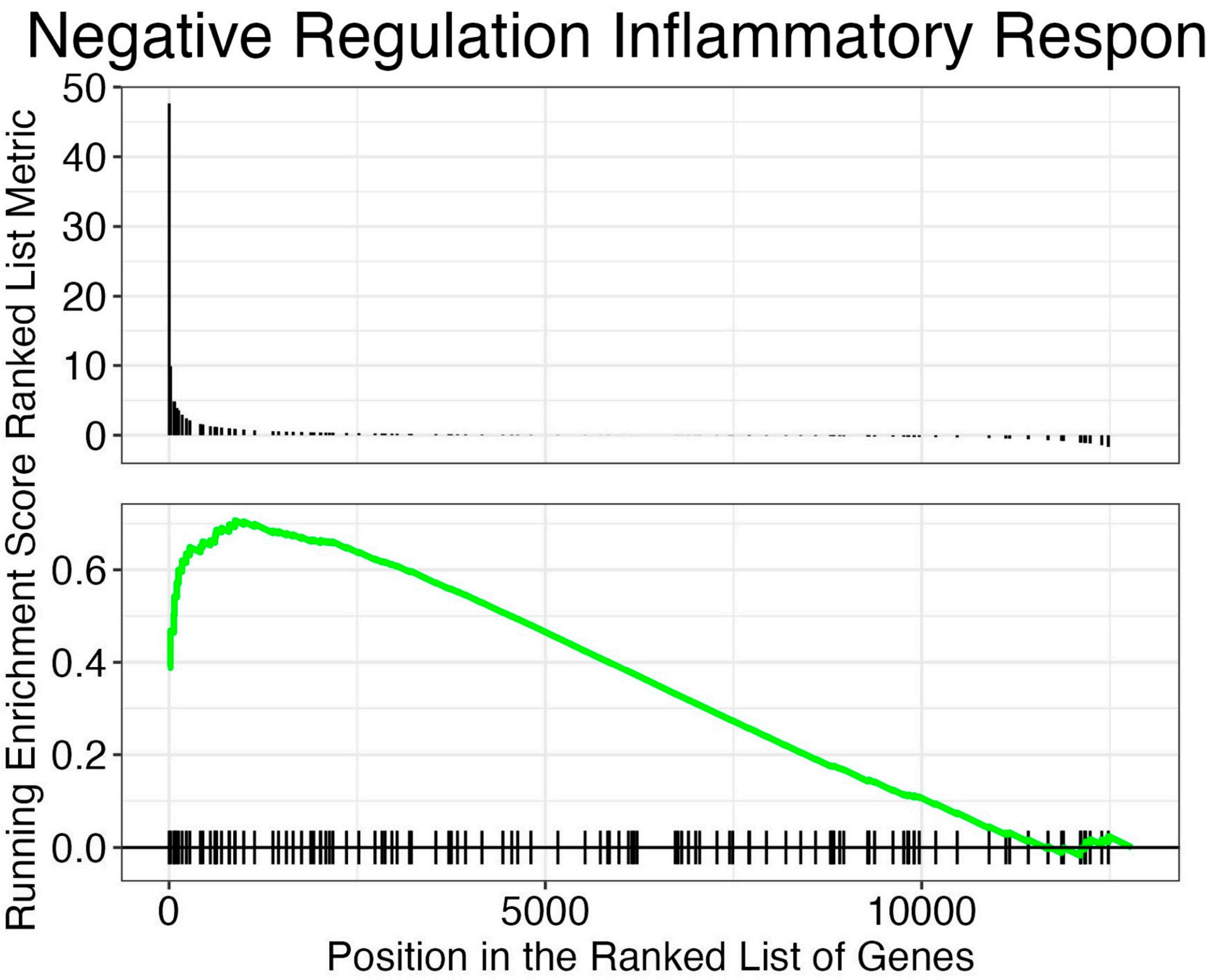
Negative Regulation Inflammatory Respon
Running Enrichment Score Ranked List Metric
50
40
30
20
10
0
0.6
0.4
0.2
0.0
0
5000
10000
Position in the Ranked List of Genes

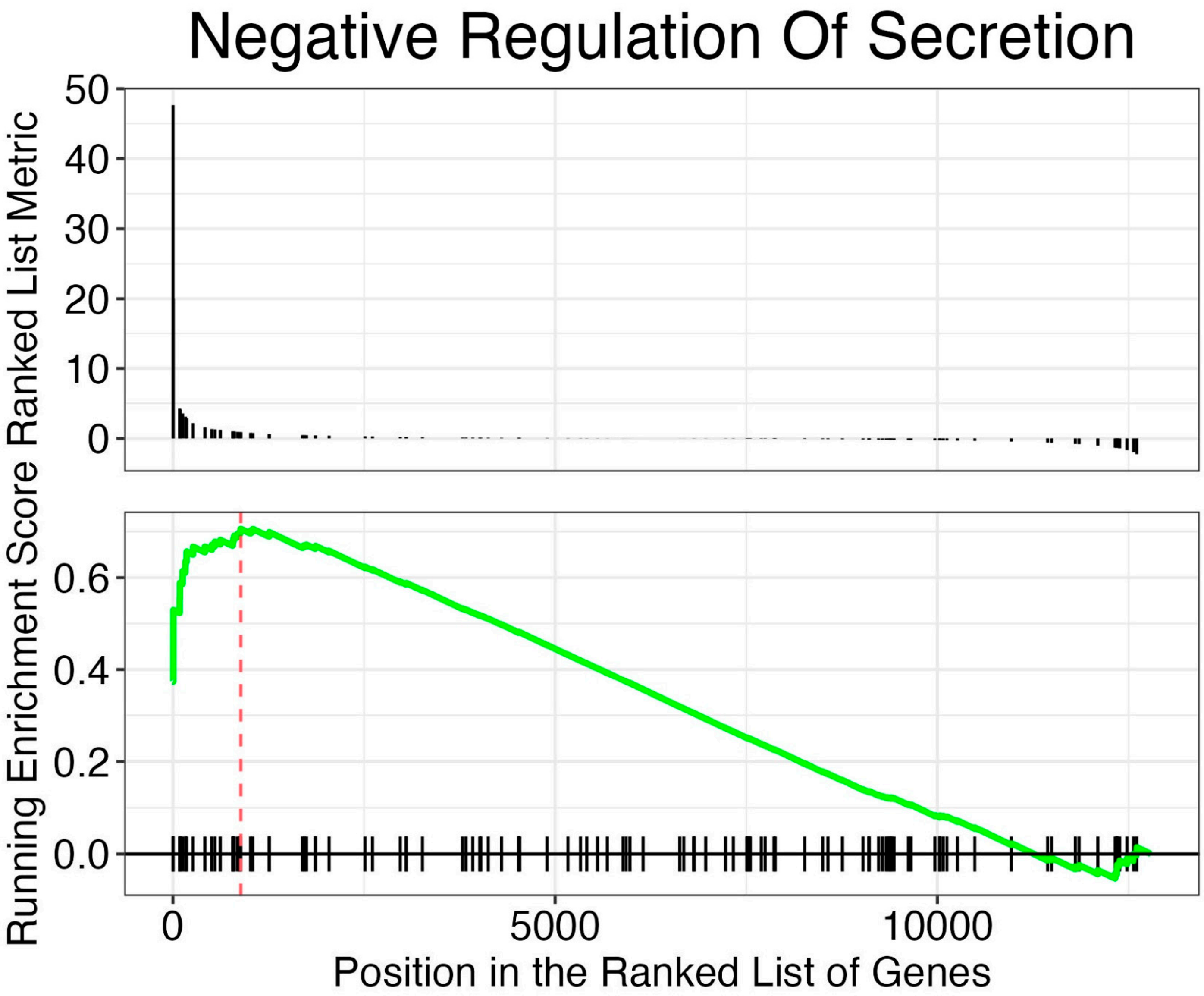
Negative Regulation Of Secretion
Ranked List Metric
Running Enrichment Score
50
40
30
20
10
0
0.6
0.4
0.2
0.0
0
5000
10000
Position in the Ranked List of Genes

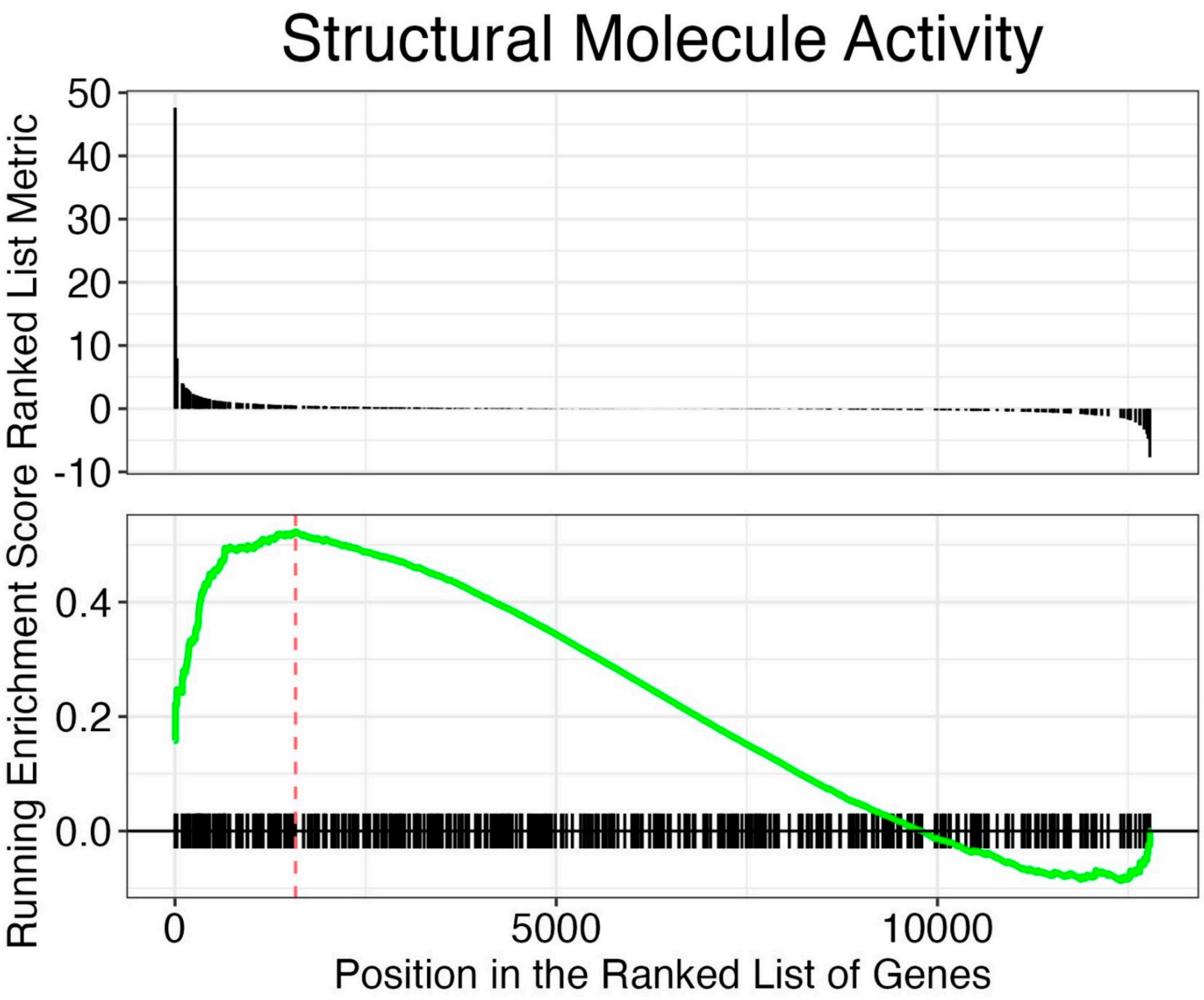

Figure S1. Gene set Enrichment analysis result (GSEA).
